# TCTA:Ir(ppy)_3_ Green Emissive Blends in Organic Light-Emitting Transistors (OLETs)

**DOI:** 10.1021/acsomega.2c04718

**Published:** 2022-11-18

**Authors:** Caterina Soldano, Ornella Laouadi, Katherine Gallegos-Rosas

**Affiliations:** Department of Electronics and Nanoengineering, School of Electrical Engineering, Aalto University, 02150Espoo, Finland

## Abstract

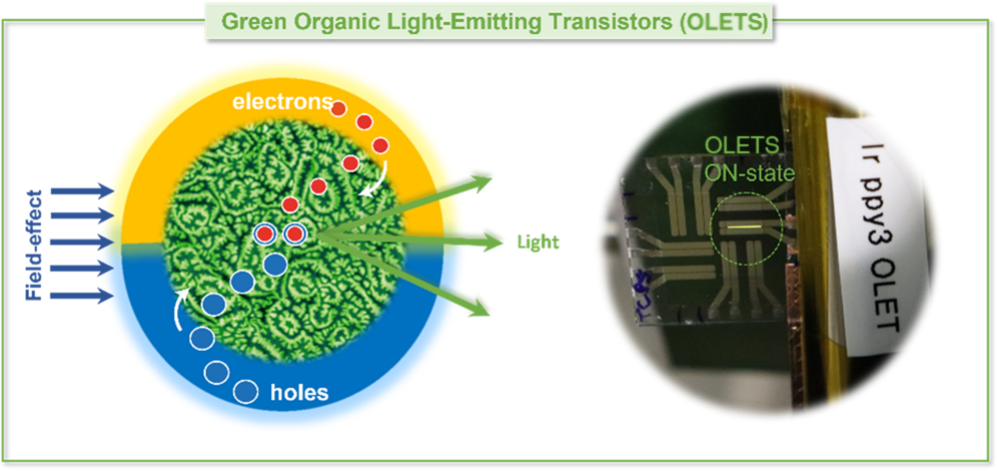

Organic light-emitting transistors are photonic devices combining the function of an electrical switch with the capability of generating light under appropriate bias conditions. Achieving high-performance light-emitting transistors requires high-mobility organic semiconductors, optimized device structures, and highly efficient emissive layers. In this work, we studied the optoelectronic response of green blends (TCTA:Ir(ppy)_3_) with varying doping concentrations in the limit of field-effect within a transistor device configuration. Increasing the dye concentration within the blend leads to a quenching of the photoluminescence signal; however, when implemented in a multilayer stack in a transistor, we observed an approximately 5-fold improvement in the light output for a 10% Ir(ppy)_3_ doping blend. We analyzed our results in terms of balanced charge transport in the emissive layer, which, in the limit of field-effect (horizontal component), leads to an improved exciton formation and decay process. While the performances of our devices are yet to achieve the state-of-the-art diode counterpart, this work demonstrates that engineering the emissive layer is a promising approach to enhance the light emission in field-effect devices. This opens the way for a broader exploitation of organic light-emitting transistors as alternative photonic devices in several fields, ranging from display technology to flexible and wearable electronics.

## Introduction

In recent years, organic light-emitting transistors (OLETs) have been increasingly gathering interest within the scientific and technological community, since they combine in the same device, the function of an electrical switch (with modulation of the channel conduction) with the capability of generating and sensing light under appropriate bias conditions.^1,2^ Multilayer OLET structures have shown higher current densities and higher external quantum efficiency (EQE), intrinsic of the device, outperforming equivalent organic light-emitting diodes (OLEDs).^3^ The light can be spatially tuned across the device channel and can thus lead to potentially both top and bottom emission. Further, the planar device structure renders OLETs ideal candidates to develop next-generation flexible displays, for which a simplified structure can be introduced at the front-plane level (light element), with an overall simplification of the manufacturing process (time, costs, yield).^4,5^

Several approaches have been proposed to achieve high-performance organic light-emitting transistors including use of high-mobility organic semiconductors (OSCs),^6,7^ high-capacitance gate dielectrics,^8^ and high-efficiency luminescent materials with high fluorescence and/or phosphorescence yield.^9^

Efficient organic light-emitting devices often exploit a vertical multilayer heterostructure, where each layer can be engineered and optimized to perform a specific function in the device, including charge injection, charge transport, and light emission. The layer responsible for the light generation can be either a single emitting material or a blend of two (or more) materials, mainly a host (matrix) and a guest (dye), the latter being the light center. Single-material emissive layers suffer from very low efficiency because of clustering and aggregation, giving rise to different quenching mechanisms (*i*.*e*. emitter–emitter interaction), thus limiting the efficiency of these dyes.^10^ Even if emitters are ultimately responsible for the light emission (exciton trapping sites), an efficient light generation process requires the presence of a host material, which has the main function of carrying the charges. These blends typically contain a small concentration (<15%) of either a phosphorescent dye or, more recently, a fluorescent TADF (thermally activated delayed fluorescence) molecule embedded in a host matrix. Many studies in the literature show that the photoluminescence signal decreases with increasing dye concentration; however, the effect of guest concentration on the charge transport in these blends is much less known. Few studies in host–guest layers show a strong suppression of the carrier mobility, with a crossover between the low concentration regime, in which the guest states act as isolated trap sites, and the high concentration regime, in which direct guest–guest hopping becomes the dominant charge transport mechanism.^11^ Also, molecular superexchange can provide an alternative pathway for guest–guest transport and thus to improve the mobility (as high as one order of magnitude) near and beyond this crossover point.^12^

Metal (Ir, Ru, Pt) complexes exhibit strong phosphorescence due to the large amount of spin–orbit coupling introduced in the system by the presence of the metal ion, which leads to high triplet emission quantum yields because of the highly mixed spin character of the emitting excited states. Among many organometallic compounds, iridium complexes are a well-known class of emitters, capable of targeting emission in a broad range within the visible region. This can be achieved by tailored molecular design and synthetic approaches through the incorporation of electron-donating or electron-withdrawing groups to either stabilize or destabilize the donor or the acceptor orbitals. Ir-based emitting layers can reach very high internal efficiency (IQE) and external quantum efficiency (EQE) when implemented in devices.^13^ Excited states of these complexes are also extremely sensitive to the local environment, including temperature and polarity of solvents with an overall effect on their charge-transfer mechanism, which reflects then in variation and possibility of tuning of the emission spectrum.^14,15^ Also, host materials for phosphorescent emitters have been developed and intensively studied to accommodate different dyes and to favor efficient light generation processes. Exciplex hosts, which are hosts based on a mixture of hole transport and electron transport materials with a large energy level gap, have been recently demonstrated to enable high-efficiency OLEDs.^16^

Tris(2-phenylpyridine)iridium(III) (Ir(ppy)_3_) is a very well-known guest material to target green emission with a reported internal efficiency of almost 100%^17^ of relevance for many fields such as display technology. Tunability in light emission in Ir(ppy)_3_ and its derivatives can be achieved by functionalizing the ppy ligand with electron-donating and electron-withdrawing substituents. Given the values of highest occupied molecular orbital–lowest unoccupied molecular orbital (HOMO–LUMO) (−3.0/–5.6eV) for Ir(ppy)_3_, few host materials with a large energy gap have been proposed for highly efficient light generation in diode architectures, including TCTA (tris(4-carbazoyl-9-ylphenyl)amine), CBP (4,4′-bis(9-carbazolyl)–1,1′-biphenyl), and mCP (*N*,*N*′-dicarbazolyl-3,5-benzene). Energy level alignment and chemical affinity between the host and the guest are key factors in multilayer structures to enable efficient exciton formation and radiative decay.^18^ External quantum efficiency as high as approximately 24% was obtained in a vertical structure exploiting a very ultrathin emissive layer (<2 nm) within an optimized OLED architecture.^19^ Li et al. have shown, for example, that Ir(ppy)_3_ can improve the injection and transport properties in TCTA films in photovoltaic architectures with a current density enhancement for increasing doping concentration.^20^

Most studies on Ir(ppy)_3_ properties are related to the optical response of the individual dye (in solution, thin film, or in blended systems using several hosts) or to the optoelectronic properties of light-emitting devices, mainly diodes, using such layers. In the case of light-emitting transistors, many considerations on energy levels and charge transport are likely to hold (at least partially); however, the effect of dye content on the light generation process in the presence of external fields is still yet not well studied and understood. In fact, while reports on organic light-emitting diodes might help elucidating some aspects related to the nanoscale transport along the vertical direction, most is unknown about the effect of the additional horizontal component in a field-effect device.

In this work, we report the electrical and the optical response of organic light-emitting transistors using TCTA:Ir(ppy)_3_ blends with different dye doping concentrations as a host–guest emissive layer within the organic stack. While we observed a quenching of the photoluminescence signal for increasing guest concentration, we found that, when implemented in the device architecture, a TCTA:Ir(ppy)_3_ blend with 10% dye content shows the largest light output. This, in the limit of the same holes and electron currents, also indicates the most efficient device. We discuss the optoelectronic response of our devices in the framework of field-effect transport, where the additional horizontal component of the field along with a balanced transport within the emissive blend plays an important role in the exciton formation and decay process.

## Results and Discussion

### Photoluminescence of TCTA:Ir(ppy)3 Blends

**Figure 1:**
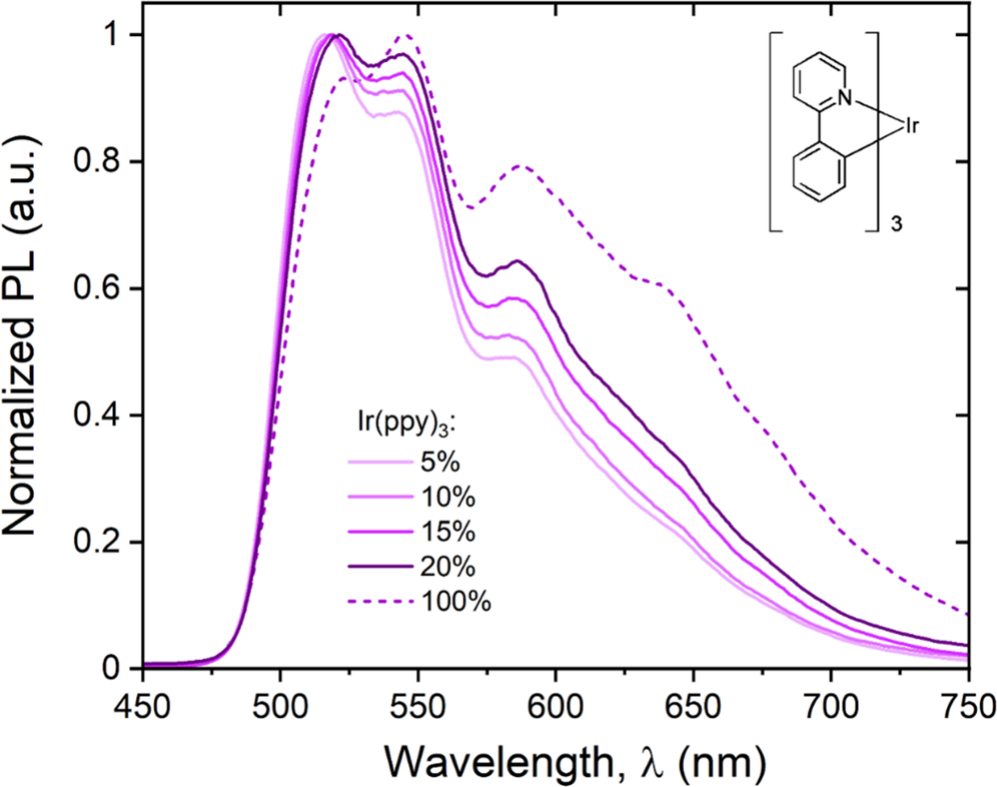
Photoluminescence of TCTA:Ir(ppy)_3_ blends. Room temperature-normalized photoluminescence (PL) excited at 403 nm for different TCTA:Ir(ppy)_3_ blends, with doping concentration labeled accordingly. The structure of the Ir(ppy)_3_ molecule is also shown.

Figure 1 shows the normalized photoluminescence (PL) spectra for the emissive layers deposited on a quartz substrate, containing different Ir(ppy)_3_ concentrations (layer thickness of 60 nm). The photoluminescence spectrum for a neat Ir(ppy)_3_ film (30 nm) is also included as reference (dash line). The overall shape of the photoluminescence spectrum is consistent with previously reported emission for Ir(ppy)_3_ and Ir(ppy)_3_ blends.^21^ All spectra show a broad emission due to strong charge-transfer (CT) characteristics of Ir-based cyclometalated complexes, with the main peak at around 515 nm, consistent with phosphorescence signal in Ir(ppy)_3_ solution.^22^ Further, we also observed two clear contributions originating from the guest at 545 and 585 nm.

As it occurs in other metal complexes (*i*.*e*., ruthenium), the lower energy excited states in Ir(ppy)_3_ result from metal-to-ligand charge-transfer (MLCT) states,^23^ according to which, upon excitation, an electron moves from the 5*d* orbital of the Ir ion out onto the p orbitals of the ligands. Spin–orbit coupling in Ir(ppy)_3_ complexes thus favors a mixture of both singlet and triplet states with approximately 70 states in the first eV.^24^ The dynamics of such excited states (crossing from the singlet ^1^MLCT state to spin-mixed ^3^MLCT state) is governed by intersystem crossing (ISC), resulting in a very fast process (∼hundreds of fs).^22^ For increasing Ir content within the blend, we observed (i) a main dominant peak around 515 nm in all blends, showing an overall redshift (Δλ ∼ 6 nm between 5 and 20%), (ii) a second peak at 545 nm, characterized by an increase in the PL signal, and (iii) a third peak around 582 nm, showing both a redshift (Δλ ∼ 5 nm between 5 and 20%) and an increase of normalized PL.

Table 1 summarizes the characteristic feature of these peaks according to their vibronic transition and spin state, as extracted directly from Figure 1. The main peak (ν (0,0)) corresponds to the fundamental band (between the ν = 0 vibrational of S_0_ to S_1_) and the ligand-centered singlet (^1^LC) state. The second at 545 nm (ν (0,1)) and third at 582 nm (ν (0,2)) peaks can be assigned as ν = 0 of S_1_ to ν = 1 of S_0_ vibronic transition, ν = 0 of S_1_ to ν = 2 of S_0_ vibronic transition, and the metal-ligand charge-transfer singlet and triplet states (^1^MLCT and ^3^MLCT), respectively. It is also important to note here that previous work also suggested that the higher wavelength peak can be a mixture of the ^3^LC and ^3^MLCT states (rather than just ^3^MLCT states).^25,26^

**Table 1:** Photoluminescence Peaks Contributing to the Emission of the Blend as a Function of the Ir(ppy)_3_ Content; See Main Text for More Details

	1st peak ν (0,0)		2nd peak ν (0,1)		3rd peak ν (0,2)		FWHM*
Ir(ppy)_3_ doping (%)	λ (nm)	PL* (a.u.)	λ (nm)	PL* (a.u.)	λ (nm)	PL* (a.u.)	(nm)
5	515.9	1	542.7	0.88	582.2	0.49	72.7
10	518	1	544.4	0.91	582.9	0.53	94.2
15	519	1	544.4	0.94	584.9	0.58	101.7
20	521.3	1	544.4	0.96	585.6	0.64	107.8
100	522.7	1	544.7	1	587.3	0.79	154.8

* Refer to normalized photoluminescence values.

We found a redshift for increasing Ir(ppy)_3_ doping concentration (see the Supporting Information, Figure S2), which we believe is due to either a non-negligeable contribution from the TCTA fluorescence signal or the effect of the increasing clustering of the dye.^27^ A similar shift has been found in CBP:Ir(ppy)_3_ blends (4 nm at 10% doping) and ascribed to exciton–exciton annihilation (two excitons meet and form a single higher excited state, leading to exciton loss, thus deviating from the original peak), which depends on the concentration of excitons.^28^ This also induces an increased interaction probability among excitons, leading to faster decay. In our current experimental conditions, we are not capable of distinguishing among these contributions.

Further, we observed an overall broadening of the spectrum for increasing dye concentrations, which is expected with self-quenching interactions between Ir(ppy)_3_ molecules at increased dopant concentrations.^17^Table 1 also includes the full-width half-maximum (FWHM) values extracted directly from data in Figure 1, which are similar to previously reported studies (>70 nm).^25^ FWHM monotonic increase with the increasing doping concentration is consistent with the strong CT characteristic of Ir(ppy)_3_.

When excited at 403 nm, both the host and guest absorb the incident radiation, reaching an excited state; we note here that any major PL contribution in the range 370–420 nm arising from the host^16^ is mainly removed from the final spectra by means of a 450 nm long-pass filter used to isolate from the laser signal. Absolute intensity of the emission shows a large decrease as the Ir(ppy)_3_ concentration increases, which, in the limit of the same optical excitation and integration times (0.5 s), directly reflects the effect of the guest content (see the Supporting Information, Figure S1). Further, it has also been shown that increasing the dye concentration will also decrease the PL quantum yield (PLQY, which is a measure of the emitted photons for incident photon); in fact, Gao et al. have demonstrated that TCTA blends with Ir concentration 1–10% have an extremely high PLQY (>80%), which decreases further with increasing guest doping. This is also accompanied by increased exciton quenching for concentrations larger than 10%.^27^

Photoluminescence quenching in host–guest systems arises mainly from exciton–exciton interactions. Several studies have recently demonstrated that increasing the guest concentration in the blend can lead to different phenomena such as (a) enhanced intermolecular interactions (exciton loss of about 23 (43)% for 20 (50)% doping) and (b) formation and growth of nonisolated Ir(ppy)_3_ clusters, leading to percolation paths. For the latter, it has been shown that for a 10% Ir content, approximately 70% of the clusters are part of interconnected clusters containing three or more molecules, while for 20% doping, it is estimated that only 5% of the molecules are not part of a larger cluster of percolation path for Ir(ppy)_3_.^27^

### Electroluminescence in Organic Light-Emitting Transistors Using TCTA:Ir(ppy)_3_ Blends

**Figure 2:**
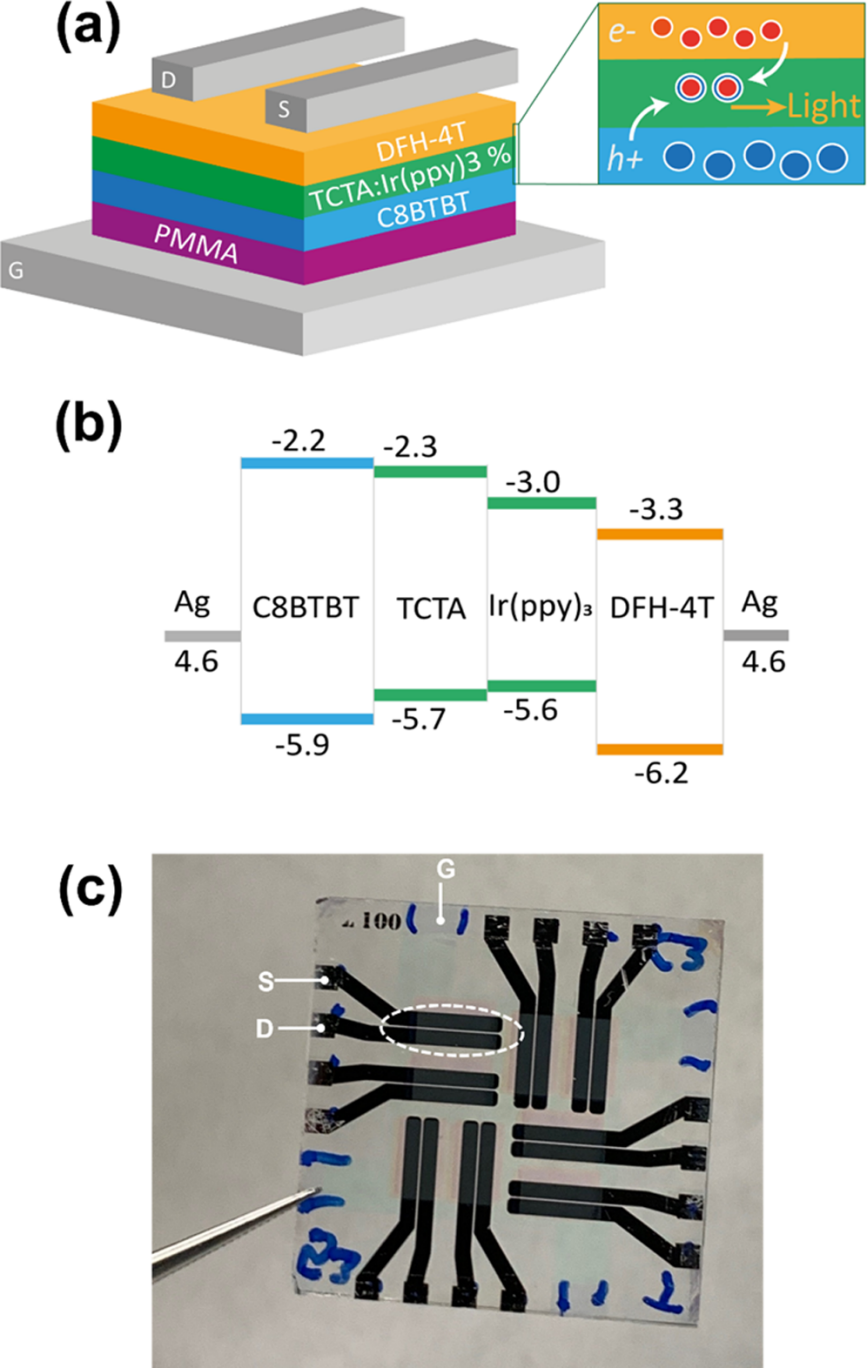
Organic light-emitting transistor: device structure and energy configuration. (a) Simplified schematic and (b) energy levels for the OLET structure. (c) Optical image of a representative glass/ITO substrate containing eight different light-emitting transistors. Electrodes are also indicated (see the manuscript for details).

We used a bottom-gate/top-contact (BG-TC) transistor configuration, as schematically shown in Figure 2a. Gate dielectric is a poly(methyl-methacrylate) (PMMA) layer with a thickness of approximately 430 nm, deposited on top of a transparent gate electrode, *G* (indium tin oxide, ITO). The device active region consists of three stacked organic layers: the first (in direct contact with the PMMA) and the third layers are field-effect hole-transporting (2,7dioctyl[1]-benzothieno[3,2-*b*][1]benzothiophene, C8BTBT, 30 nm, Sigma-Aldrich) and electron-transporting (α,ω-diperfluorohexyl-quarterthiophene, DFH-4T, 45 nm, Sigma-Aldrich) semiconductors, respectively, whereas the intermediate layer, where the electron–hole recombination and light generation processes occur, is a host–guest matrix system. The emissive layer is a blend (60 nm) of TCTA and Ir(ppy)_3_, with different doping concentrations (both materials from AmericanDyeSource Inc.). Drain (D) and source (S) electrodes (silver, 70 nm) are then deposited on top of the uppermost organic layer. Transistor channel length (*L)* and width (*W)* are 100 μm and 5 mm, respectively. Figure 2b shows the energy diagram of the organic multilayer stack, where the *n*- and the *p*-type organic semiconductors provide electrons and holes to the emissive layer under the effect of the external field, respectively. We refer the reader to (3, 29) for general considerations on the energetics of the multilayer heterostructures and materials therein. Figure 2c shows an optical image of one of our representative substrates, containing eight different transistors and a common gate with electrodes labeled accordingly.

**Figure 3:**
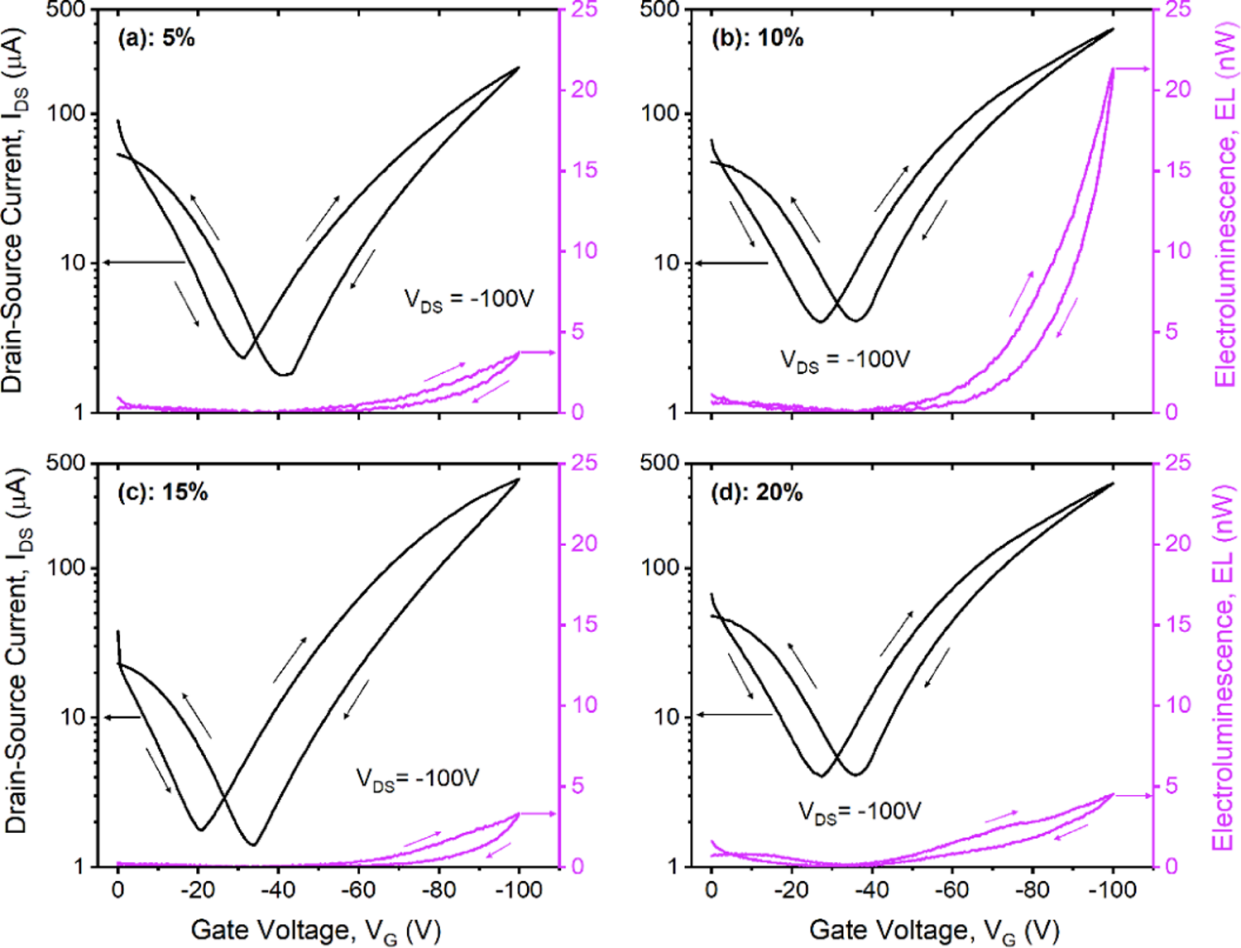
Optoelectronic characterization of OLET with different TCTA:Ir(ppy)_3_ blends. Saturation transfer curves (*I*_DS_*vsV*_G_, with *V*_DS_ = −100 V) and optical output (EL, bottom emission) for organic light-emitting transistors using (a) 5%, (b) 10%, (c) 15%, and (d) 20% Ir(ppy)_3_ doping concentration within the blend. Drain-source voltage (*V*_DS_) and sweep directions are also indicated in each panel.

Figure 3 shows the optoelectronic characterization of organic light-emitting transistors using TCTA:Ir(ppy)_3_ blends with same thickness but different Ir contents of (a) 5%, (b) 10%, (c) 15%, and (d) 20%. The rest of the structure is the same for all devices, and it has the same geometrical features (L, W). The electroluminescence (EL) signal reported on the right *y*-axis refers to the light output extracted through the transparent gate electrode (bottom emission) with a photodiode in direct contact with the substrate with same scale in all panels for a more direct comparison.

Our experimental results show that all transistors are operating in an ambipolar regime, as suggested by the typical “*V*” shape of the *p*-transfer curve in all panels. Devices are dominated by hole transport, which is approximately less than 1 order of magnitude larger than the electron contribution, leading to an overall balanced charge transport in the device within the range of applied bias. In the case of an ambipolar multilayer transistor, the operation of the device can be seen as two parallel organic thin-film transistors, each one carrying one type of charges. During the *I*_D_*–V*_G_ transfer sweep (any panel in Figure 3), the light is generated either (a) when only holes are moving within the device (right side of the black curve) or (b) when both transistors are in their ON-state and charge density distributions are relatively balanced (in the vicinity of the curve apex) and where an increasing number of minority charge carriers from the *n*-type semiconductor layer are injected toward the recombination area. As a result, in a (organic light-emitting) transistor operating in an ambipolar regime, the drain-source current in the saturation region can be described as
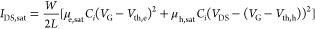
1where *C*_*i*_ is the capacitance per unit area of the dielectric layer (*C_i_* (PMMA) = 6.6 nF/cm^2^), μ_sat_ and *V*_th_ are the device saturation mobility and threshold voltages for holes (h) and electrons (e), and *W* (5 mm) and *L* (100 μm) are the transistor channel width and length, respectively.

Table 2 summarizes the optoelectronic properties of OLETs using TCTA:Ir(ppy)_3_ blends with different Ir(ppy)_3_ contents. We observed no specific dependence from the Ir(ppy)_3_ doping concentration for both currents (at the highest applied bias *V*_DS,max_ = *V*_G,max_ = |100| V); however, we noted a larger variation for the holes compared to the electron counterpart (see Figure S3 in the Supporting Information). Field-effect saturation mobility and threshold voltage for holes and electrons are calculated from the linear fit of  from the forward sweep of the locus curves (*I*_DS_*vsV*_G_ = *V*_DS_, Figures S4 and S5 in the Supporting Information). We found values of hole (electron) mobilities of 0.4–1 (0.1–0.25) cm^2^/Vs (see Table 2) with seemingly no clear dependence from the doping concentration of the emissive blend (see later in the manuscript). These values are less than 1 order of magnitude smaller compared to the corresponding single-layer organic field-effect transistor based on each semiconductors; for this, we refer the reader to one of our recent studies using the same set of materials.^30^ Threshold voltages are found in the range of −|40–50| and 49–52 V for holes and electrons, respectively. These values are consistent with organic light-emitting transistors using low-k PMMA as a dielectric layer,^31^ and no correlation with the doping concentration is found. Larger variation in the case of the holes might be due to interfaces, which can affect the charge injection and transport within the stack. All devices show negligible gate leakage currents of approximately a few tens of nA within the range of our applied bias (at least 4 orders of magnitude lower than the maximum values of the drain-source current, *I*_DS,max_).

**Table 2:** Optoelectronic Properties of the Device Using Different TCTA:Ir(ppy)_3_ Blends^a^

		Ir(ppy)_3_ content (%)			
		5	10	15	20
saturation mobility, *μ_sat_*(cm^2^/Vs)	h^+^	0.44	0.89	0.97	0.78
	e^–^	0.24	0.18	0.10	0.11
threshold voltage, *V_th_* (V)	h^+^	–45	–49.3	–41.9	–39.5
	e^–^	49.6	52.4	49.3	50.4
maximum drain-source current, *I*_DS-max_ (μA)	h^+^	205	380	397	365
	e^–^	75	58	31	65
gate current, *I*_G_ @V_DS,max_ (nA)		∼10s			
optical power, *EL* @V_DS,max_ (nW)		3.7	21.4	3.3	4.5
external quantum efficiency, *EQE* @V_DS,max_ (×10^–3^%)		0.8	2.5	0.4	0.6

a Summary of the figure of merit of the organic light-emitting transistors using different TCTA:Ir(ppy)_3_ blends. Values refer to the device operating in a saturation regime and, when relevant, are reported for both holes and electrons.

In ambipolar organic light-emitting transistors, the light is expected to be originated in the channel area, close to the drain electrode, where exciton recombination can be maximized through efficient electron–hole balancing. A decreasing degree of ambipolarity will move the site of the light generation to the proximity of the drain electrode, where charge–exciton quenching might prevent efficient recombination processes and where a nontransparent electrode might hinder light extraction in that direction. Moreover, very recently Moschetto et al. have revealed that in multilayer OLETs, it is also indeed possible to achieve light emission within the channel even if the electrical characteristics of the device might indicate a dominating unipolar regime.^32^ This is because light emission (and efficiency) also depends on the injection of the charges from their respective layer toward the emissive layer, not necessarily reflected by an unbalance between field-effect currents; in this sense, vertical fields (gate field) can play a role in promoting charges toward exciton formation sites.

Measured light output in our OLETs (Figure 3) represents only a part of the total light generated in the device, while it is of our interest to measure the portion of the light extracted also from the top of the device; this is currently beyond our experimental capabilities.

We observed also a non-negligible hysteresis in our devices with a constant shift of approximately 8–9 V toward higher bias with the level of source–drain current remaining almost unchanged (apex is considered as a reference point). This hysteretic behavior in the charge transport is also reflected in the light output with no clear dependence from the Ir(ppy)_3_ content. A detailed investigation of this effect is currently ongoing, and we expect this to be related to different charge transport and injection regimes (holes *vs* electrons) and possible charge trapping localized at the dielectric interface.^33^

**Figure 4:**
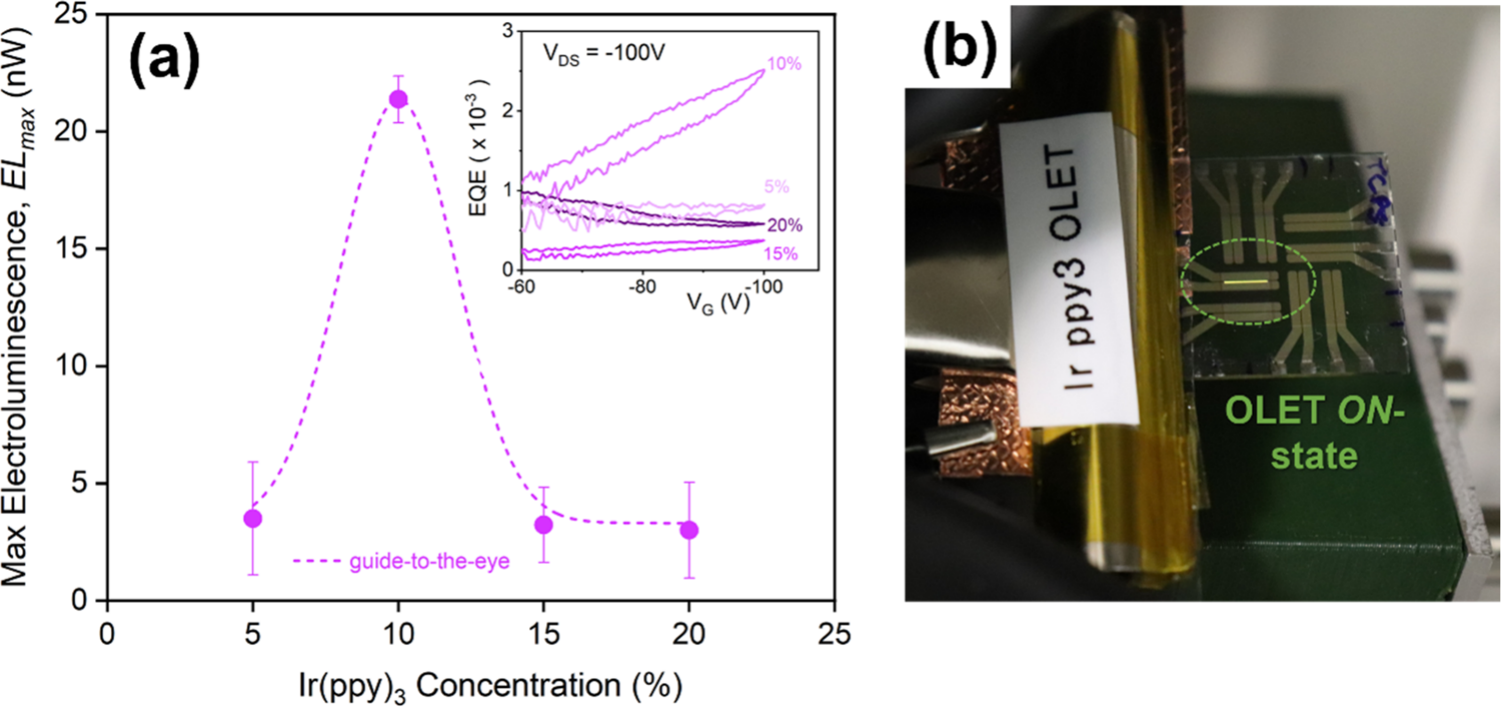
Electroluminescence of OLETs with different TCTA:Ir(ppy)_3_ blends. (a) Maximum electroluminescence (EL_max_) and (inset) external quantum efficiency in OLET as function of the Ir(ppy)_3_ concentration. Dashed line is a guide-to-the-eye only. (b) Optical image of an organic light-emitting transistor (TCTA:Ir(ppy)_3_ 10% blend) in its ON-state, showing the characteristic green emission over the entire channel.

Figure 4a summarizes the electroluminescence of the OLET devices using different TCTA:Ir(ppy)_3_ blends, which in the limit of similar electrical characteristics (hole and electron currents), clearly demonstrates that the blend with 10% Ir content produces the largest optical output when implemented in a transistor configuration. For such concentrations, we observed an increase of an approximate factor of 5 in the light output compared to other blends. Reported electroluminescence values are averaged among several devices with the same dye concentration on the same substrate.

The inset of Figure 4a shows the external quantum efficiency of the devices with different emissive blends in a saturation regime. All EQE values (except the one with 10% Ir content) are nearly independent from the gate bias, while for TCTA:Ir(ppy)_3_ 10% emissive layer, the efficiency is seemingly linearly increasing with the gate voltage (field). While device ambipolarity ensured the injection of approximately the same number of charges, such a transport regime does not necessarily ensure an efficient exciton radiative decay, for which other features such as improved interfaces, enhanced charge injection, or transfer in the emissive layer^34^ can play an important role in the exciton formation and dynamics. Figure 4b shows a representative optical image of an organic light-emitting transistor fabricated on a glass/ITO substrate using 10% blend as an emissive layer, while in its ON-state shows the Ir(ppy)_3_ characteristic green emission along the entire channel.

As already mentioned, the measured light is only a part of the total light generated in the device, so the EQE values are expectedly underestimated.^35^ We note that devices using these blends are still far from being comparable with corresponding values in state-of-the-art organic light-emitting devices, including diodes.

Prosa et al. used a similar set of materials in OLETs to investigate how different injection layers, responsible for carrying the electrons toward the emissive layer, can affect the overall device performance. In this work, only one blend composition (TCTA:Ir(ppy)_3_ 20%) was used and deposited on C8BTBT (similarly to this work), and EQE values of 0.11 and 0.02% for OLET were obtained with and without an EIL, respectively. While we can only speculate that this doping concentration might represent one of the authors most efficient configuration, we observe that in their case, OLETs are operating in an unipolar regime, suggesting that the measured bottom emission might also include a contribution from the light reflection from the drain electrode. Further, a different channel geometry factor (W/L) might affect the current level (see eq 1) and the corresponding light output; in fact, Prosa et al. showed a *W*/*L* (=12 mm/70 μm) value, which is about 3.4 times that in our devices (=5 mm/100 μm).^36^

Other studies reported Ir(ppy)_3_ blended with CBP, a common host, which, similarly to TCTA with a large energy gap, can easily favor energy transfer in the host–guest system and thus efficient light emission process in Ir(ppy)_3_ blends. McCarthy et al. exploited this blend in the so-called vertical-OLET (v-OLET) configuration to achieve green light emission in low-current-driven device with an approximate luminance of 1000 cd/m^2^.^37^ It is however challenging to compare our results with the ones from McCarthy et al.; in fact, while a v-OLET shows a degree of conductance (and light) modulation, it is not strictly speaking a field-effect device since the structure comprises a capacitor cell coupled with an OLED. In this sense, the dynamics of the charge transport responsible for the light emission is still dominated along the vertical direction instead of horizontally as in the case of the present work.

Further, we also investigated the morphology of the emissive layer within the device stack. In fact, it is known that for the very same material(s), molecular packing and intermolecule interaction can strongly affect the electrical and optical properties of the material itself.^38^ We here recall that the blends for optical studies (Figure 1) have been fabricated at the same time as the ones integrated in the OLET device (Figures 3 and 4). On the other hand, the blends have been deposited on different surfaces: directly on quartz for optical studies and on a C8BTBT film for the OLET fabrication (on a PMMA dielectric layer).

**Figure 5:**
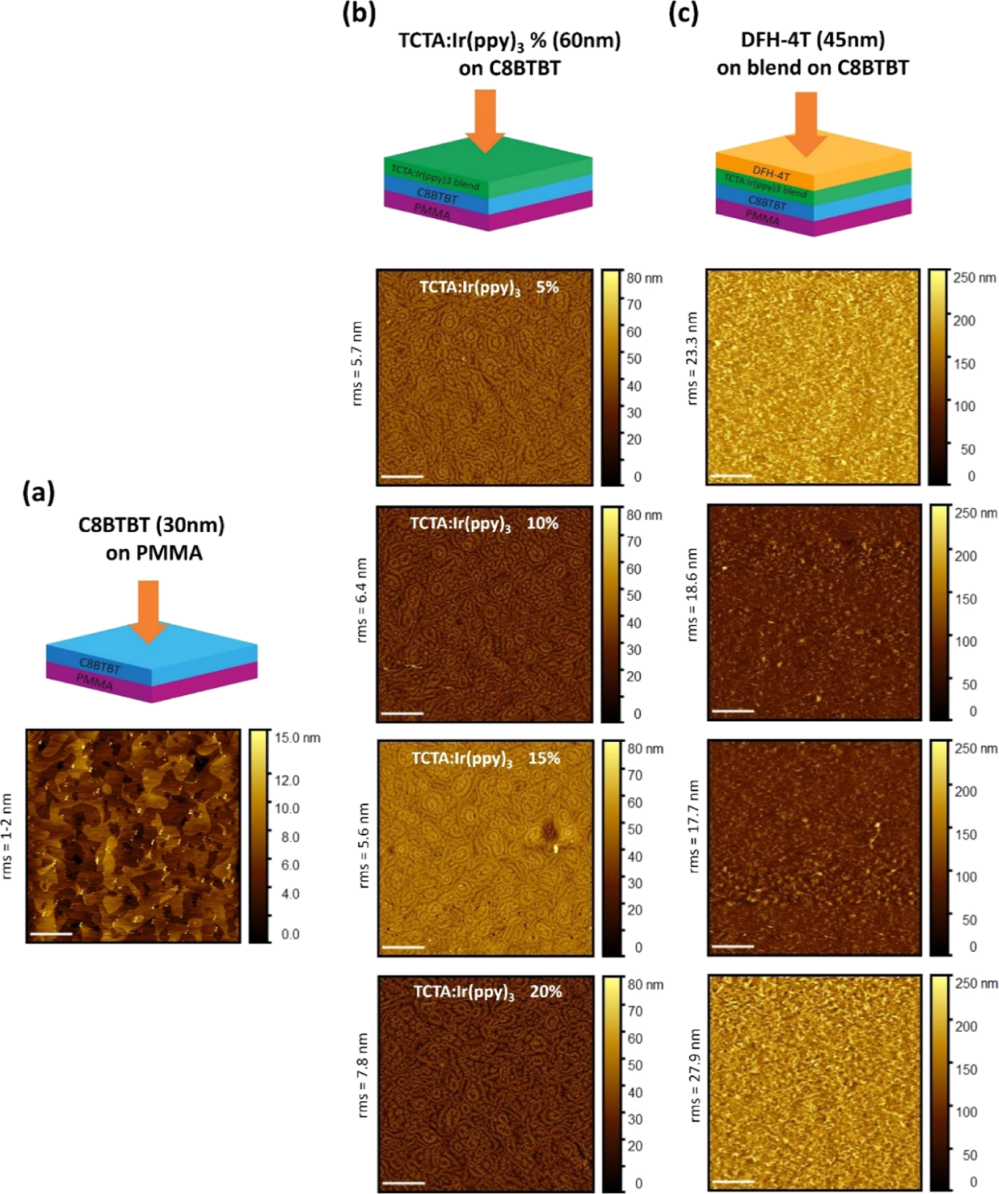
Surface morphology of different organic films within the multilayer stack. Atomic force microscope images (AFM) of single organic layers within the multilayer stack (left to right, according to schematics simplified on the top): (a) C8BTBT on PMMA, (b) TCTA:Ir(ppy)_3_ blends with different guest concentrations on C8BTBT, and (c) DFH-4T deposited on the blends. Image size (10 μm × 10 μm) and scale bar (2 μm) are the same for all micrographs.

Figure 5 shows the surface structure of each organic layer within the organic stack investigated by atomic force microscopy (AFM). Schematics on top of each column indicate the imaged topmost layer. The scan size is 10 μm × 10 μm, and the scale bar is 2 μm for all micrographs. Figure 5a shows the typical island-like structure of C8BTBT grown on the PMMA layer (rms <1 nm),^30^ characterized by an extremely flat surface (rms <2 nm), consistent with the growth mechanism reported in the literature.^33^ The C8BTBT film exhibits relatively large grain sizes and good connectivity, which is then reflected in the overall high hole mobility value even in the multilayer heterostructure. Further, deposition of the TCTA:Ir(ppy)_3_ blend seems to retrace the underlying C8BTBT surface, as shown in panel (b). The observed morphology is very similar to one of our previous works using TCTA blends with another Ir-based dye (Ir(piq)_3_ for red emission), deposited on the same *p*-type layer.^39^

All Ir(ppy)_3_ blends show very similar structures and values of surface roughness (5–8 nm) with no clear dependence from the Ir content. When the topmost organic layer (DFH-4T) is further deposited (panel (c)), the blend surface and its structure appear to be fully buried with an overall increased surface roughness (rms ∼18–28 nm) and no correlation with the guest concentration. A rough DFH-4T surface can enable a more efficient charge injection from electrodes evaporated on top. In summary, our morphological analysis of different interfaces shows no major differences among the active organic stack.

Based on our experimental findings and considerations so far, we then suggest that the improved light output (and efficiency) in the case of the blend with 10% Ir content can result from (a combination of) the following:

(a) Purely optical response of the emissive layer: in fact, TCTA:Ir(ppy)_3_ blends with Ir content up to 10% exhibit a high PL quantum yield, PLQY (>80% independent of the doping concentration in the range 1–10%). Further increasing the guest concentration leads to a drastic decrease of the PLQY (about 60% for the Ir concentration of 20%).^27^
(b) Enhanced electron transport of the blends, which leads to an overall well-balanced charge transport in the emissive layer. At room temperature and in the limit of the same applied field, a mobility of approximately 10^–6^ cm^2^/Vs was found for both holes and electrons in thick TCTA:Ir(ppy)_3_ blends (>1 μm) with Ir content of 7% with time-of-flight (ToF) experiments.^40^ Extrapolating values of mobility for fields like the ones applied to OLET devices, in this work, showed an improvement of almost an order of magnitude can be expected. Gao et al. have recently demonstrated through metal–insulator–semiconductor charge extraction by linearly increasing voltage (MIS-CELIV) studies that a 10% Ir doping leads to a balanced charge transport also in thin TCTA:Ir(ppy)_3_ blends (mobility ∼10^–6^ cm^2^/Vs). Holes are primarily transported by the TCTA and electrons from the Ir complex with hole mobility of a neat TCTA film significantly reduced with increasing Ir(ppy)_3_ concentration. Blends with higher doping show similar hole and electron mobilities, suggesting that transport of both charges occurs primarily at the guest molecule sites. This likely results upon the formation of Ir(ppy)_3_ percolation pathways for hole transport^27^ and then decrease the number of traps.^41^ Many electroluminescence studies in host–guest systems are performed in the limit of applied vertical fields (*i.e.* capacitor-like structure such as electrode/blend/electrode) of relevance for organic light-emitting diode operation. However, in the case of a light-emitting transistor, the active organic stack is subjected to both vertical and horizontal fields. Upon biasing of the device and polarization of dielectric layers and interfaces, charges accumulate within a thin layer (∼few nanometers) and the transport occurs mainly along the horizontal direction (across the channel over tens of micrometers). With charges (and excitons) traveling for such long distances under the effect of external fields, charge transport properties of the host become important for an efficient charge and energy transfer process to take place. Also, the vertical component of the field can partially extend their motion within the blend and, at the same time, improve the vertical injection of charges toward the emissive layer. Thus, an overall balanced transport in the layer (as in the case of the TCTA:Ir(ppy)_3_ 10% blend) can lead to a more favorable and efficient charge transfer in the presence of an external field (*i*.*e*. changes in recombination rates, radiative, and nonradiative as well as reduced quenching phenomena), especially for electrons toward the dye molecule sites.^40^ This is also supported by recent simulations, according to which in a 10% doped TCTA and Ir(ppy)_3_ blend, about 70% of all Ir molecules are part of interconnected clusters containing three or more molecules, and conditions compatible with charge hopping and Dexter energy transfer among adjacent complexes.^27,42^
(c) Exciton quenching, including exciton–exciton quenching as well as charge–exciton quenching. In particular, for the case of Ir(ppy)_3_, it is known that solid state blends suffer from a strong contribution from triplet–triplet annihilation (TTA), for which efficient triplet migration occurs through exciton hopping in locally dense clusters of emitter molecules.^43,44^ Thus, light output and performances in organic light-emitting devices are limited by self-quenching mechanisms among guest molecules and clusters occurring at larger dopant concentrations within the blend. Electric-field assisted exciton dissociation might also play a role for the electroluminescence quenching, particularly at high biases and concentrations, affecting the amplitude or the quenching rate mechanisms.^45,46^ In this work, only the gate field exceeds 1MV/cm, which might considerably increase the exciton dissociation efficiency but not the horizontal one. As for the charge–exciton quenching, from a macroscopic point of view, this can result from the interaction of the excitons with charges closer to the (drain) electrode since. Further, at higher biases, our devices are dominated by a hole transport regime, with light emission localized closer to the drain electrode, where the exciton light could be partially quenched due to the presence of the electrode.^47−49^ Excited state annihilation can also occur upon interaction with free or trapped charge carriers, where it might be relevant to distinguish between holes and electrons, since corresponding annihilation rates might be different.^50,51^

These mechanisms play a role in the performance of organic light-emitting transistors using TCTA:Ir(ppy)_3_ blends as an emissive layer; however, identifying and quantifying each individual contribution is currently beyond our experimental capabilities and the scope of the present manuscript.

## Conclusions

In this work, we studied how the doping concentration in the emissive blend based on a TCTA:Ir(ppy)_3_ blend affects the performances of organic light-emitting transistors. From a purely optical point of view, increasing the dye concentration within the blend leads to a quenching of the photoluminescence signal. When these blends are then implemented in the vertical stack of light-emitting field-effect devices, we observed an approximate 5-fold improvement in the light output for a 10% Ir(ppy)_3_ doping blend. We analyzed our results in terms of balanced hole and electron charge transport in the emissive layer, which, in the limit of field-effect transport, leads to an improved exciton formation and decay process.

While the efficiency of our devices is yet to achieve the state-of-the-art diode counterpart, this work demonstrates that engineering the emissive layer is a promising approach to enhance the light emission in field-effect devices. This opens the way for a broader exploitation of organic light-emitting transistors as alternative photonic devices in several fields, including display technology and flexible and wearable electronics.

## Experimental Section

### Device Fabrication and Characterization

Glass/ITO substrates were cleaned in an ultrasonic bath in diluted Hellmanex III for 10 min, deionized (DI) water for 5 min, acetone twice for 10 min, and 2-propanol for 10 min and then dried under a nitrogen flow. All substrates then underwent surface treatment with oxygen plasma (15 min, 100 W) before dielectric film deposition. PMMA (Allresist AR-P 669.06) films were fabricated by spin-coating and annealed in air on a hot plate at 110 °C for about 30 min to remove the solvent. The thicknesses of dielectric films and organic layers were measured using a stylus Dektak/XT profilometer. Surface morphology is investigated with an atomic force microscope (Bruker Dimension Icon) with a scan size area of 10 μm × 10 μm. Devices were fabricated in a Moorfield Nanotechnology MiniLab90 equipped with four LTE (low-temperature evaporation) sources for organic deposition and two TE (thermal sources) for metal evaporation. Film fabrication was carried in vacuum at a base pressure of 10^–7^ mbar. Amorphous Ir(ppy)_3_ thin-film (30 nm thick, at a rate of 0.15 Å/s) and TCTA:Ir(ppy)_3_ blends (60 nm) were deposited on a precleaned quartz substrate in vacuum at a base pressure of 5 × 10^–7^ mbar. The deposition rate for the host was kept constant (1 Å/s) for all blends. Electro-optical characteristics were measured in a glovebox at room temperature through a homemade system coupled with a B1500A Keysight semiconductor parametric device analyzer. Light output was measured with a Hamamatsu S1337 photodiode placed in direct contact with the substrate (to measure light emitted through the substrate, bottom emission).

### Photoluminescence Studies

Photoluminescence spectra have been measured with the SNOM α 300 from Witec (confocal microscope function, reflection mode, 50× objective). A purple laser was used (excitation wavelength at 403 nm, incident power 0.6 mW, integration time 0.5 s, and long-pass filter at 450 nm).

## Supporting Information Available

Photoluminescence of TCTA:Ir(ppy)_3_ blends (Figure S1); normalized photoluminescence of TCTA:Ir(ppy)_3_ blends (Figure S2); hole and electron currents in organic light-emitting transistors (Figure S3); and locus curves (p- and n-type) of OLET devices with different TCTA:Ir(ppy)_3_ blends (Figures S4 and S5) (PDF)

## References

[ref1] Muccini M.; Koopman W.; Toffanin S. The photonic perspective of organic light-emitting transistors. Laser Photonics Rev. 2012, 6, 258–275. 10.1002/lpor.201100008.

[ref2] Zaumseil J. Recent Developments and Novel Applications of Thin Film, Light-Emitting Transistors. Adv. Funct. Mater. 2020, 30, 1905269 10.1002/adfm.201905269.

[ref3] Capelli R.; Toffanin S.; Generali G.; Usta H.; Facchetti A.; Muccini M. Organic light-emitting transistors with an efficiency that outperforms the equivalent light-emitting diodes. Nat. Mater. 2010, 9, 496–503. 10.1038/nmat2751. 20436466

[ref4] Muccini M.; Toffanin S. Organic Light-Emitting Transistors: Towards the Next Generation Display Technology; John Wiley & Sons: Hoboken, NJ, USA, 2016.

[ref5] Hsieh H.-H.; Chen W.-C.; Generali G.; Soldano C.; D’Alpaos R.; Turatti G.; Biondo V.; Muccini M.; Huitema H.; Facchetti A. Flexible Active-Matrix OLET Display on a Plastic Substrate. Society for Information Display. SID Symp. Dig. Tech. Pap. 2016, 47, 739–742. 10.1002/sdtp.10744.

[ref6] Bronstein H.; Nielsen C. B.; Schroeder B. C.; McCulloch I. The role of chemical design in the performance of organic semi-conductors. Nat. Rev. Chem. 2020, 4, 66–77. 10.1038/s41570-019-0152-9. 37128048

[ref7] Okamoto T.; Kumagai S.; Fukuzaki E.; Ishii H.; Watanabe G.; Niitsu N.; Annaka T.; Yamagishi M.; Tani Y.; Sugiura H.; et al. Robust, high-performance n-type organic semiconductors. Sci. Adv. 2020, 6, eaaz0632 10.1126/sciadv.aaz0632. 32494668 PMC7195148

[ref8] Soldano C. Engineering dielectric materials for high-performance organic light emitting transistors (OLETs). Materials 2021, 14, 3756 10.3390/ma14133756. 34279327 PMC8269812

[ref9] Liu Y.; Li C.; Ren Z.; Yan S.; Bryce M. R. All-organic thermally activated delayed fluorescence materials for organic light-emitting diodes. Nat. Rev. Mater. 2018, 3, 18020 10.1038/natrevmats.2018.20.

[ref10] Zaumseil J.; Donley C. L.; Kim J.-S.; Friend R. H.; Sirringhaus H. Efficient Top-Gate, Ambipolar, Light-Emitting Field-Effect Transistors Based on a Green-Light Emitting Polyfluorene. Adv. Mater. 2006, 18, 2708–2712. 10.1002/adma.200601080.

[ref11] Yimer Y. Y.; Bobbert P. A.; Coehoorn R. Charge transport in disordered organic host-guest systems: effects of carrier density and electric field. J. Phys.: Condens. Matter 2008, 20, 335204 10.1088/0953-8984/20/33/335204.

[ref12] Symalla F.; Friederich P.; Massé A.; Meded V.; Coehoorn R.; Bobbert P.; Wenzel W. Charge Transport by Superexchange in Molecular Host-Guest Systems. Phys. Rev. Lett. 2016, 117, 276803 10.1103/PhysRevLett.117.276803. 28084749

[ref13] Adachi C.; Baldo M. A.; Thompson M. E.; Forrest S. R. Nearly 100% internal phosphorescence efficiency in an organic light-emitting device. J. Appl. Phys. 2001, 90, 5048–5051. 10.1063/1.1409582.

[ref14] O’Brien D. F.; Baldo M. A.; Thompson M. E.; Forrest S. R. Improved energy transfer in electrophosphorescent devices. Appl. Phys. Lett. 1999, 74, 442–444. 10.1063/1.123055.

[ref15] Noh Y.-Y.; Lee C.-L.; Kim J.-J.; Yase K. Energy transfer and device performance in phosphorescent dye doped polymer light emitting diodes. J. Chem. Phys. 2003, 118, 2853–2864. 10.1063/1.1535211.

[ref16] Song W.; Lee H. L.; Lee J. Y. High triplet energy exciplex hosts for deep blue phosphorescent organic light-emitting diodes. J. Mater. Chem. C 2017, 5, 5923–5929. 10.1039/C7TC01552F.

[ref17] Kawamura Y.; Goushi K.; Brooks J.; Brown J. J.; Sasabe H.; Adachi C. 100% phosphorescence quantum efficiency of Ir(III) complexes in organic semiconductor films. Appl. Phys. Lett. 2005, 86, 071104 10.1063/1.1862777.

[ref18] Sanderson S.; Philippa B.; Vamvounis G.; Burn P. L.; White R. D. Elucidating the effects of guest-host energy level alignment on charge transport in phosphorescent OLEDs. Appl. Phys. Lett. 2019, 115, 263301 10.1063/1.5131680.

[ref19] Kang S. W.; Baek D.-H.; Ju B.-K.; Park Y. W. Green phosphorescent organic light-emitting diode exhibiting highest external quantum efficiency with ultra-thin undoped emission layer. Sci Rep. 2021, 11, 8436 10.1038/s41598-021-86333-9. 33875674 PMC8055988

[ref20] Li B.; Chen J.; Yang D.; Zhao Y.; Ma D. The effects of tris(2-phenylpyridine) iridium on the hole injection and transport properties of 4,4′,4″-tri(N-carbazolyl)-triphenylamine thin films. Thin Solid Films 2012, 522, 352–356. 10.1016/j.tsf.2012.08.021.

[ref21] Seo J. H.; Han N. S.; Shim H. S.; Kwon J. H.; Song J. K. Phosphorescence Properties of Ir(ppy)3 Films. Bull. Korean Chem. Soc. 2011, 32, 1415–1418. 10.5012/bkcs.2011.32.4.1415.

[ref22] Hedley G. J.; Ruseckas A.; Samuel I. D. W. Ultrafast Luminescence in Ir(ppy)_3_. Chem. Phys. Lett. 2008, 450, 292–296. 10.1016/j.cplett.2007.11.028.

[ref23] King K.; Spellane P.; Watts R. Excited-state properties of triply ortho-metalated iridium(III) complex. J. Am. Chem. Soc. 1985, 107, 1431–1432. 10.1021/ja00291a064.

[ref24] Matsushita T.; Asada T.; Koseki S. Relativistic Study on Emission Mechanism in Tris(2-phenylpyridine)iridium. J. Phys. Chem. C 2007, 111, 6897–6903. 10.1021/jp0708796. 17149849

[ref25] Ha J. M.; Hur S. H.; Pathak A.; Jeong J.-E.; Woo H. Y. Recent advances in organic luminescent materials with narrowband emission. NPG Asia Mater. 2021, 13, 53 10.1038/s41427-021-00318-8.

[ref26] Wang H.; Liao Q.; Fu H.; Zeng Y.; Jiang Z.; Ma J.; Yao J. Ir(ppy)_3_ phosphorescent microrods and nanowires: promising micro-phosphors. J. Mater. Chem. 2009, 19, 89–96. 10.1039/B814007C.

[ref27] Gao M.; Lee T.; Burn P. L.; Mark A. E.; Pivrikas A.; Shaw P. E. Revealing the interplay between charge transport, luminescence efficiency, and morphology in organic light-emitting diode blends. Adv. Funct. Mater. 2020, 30, 1907942 10.1002/adfm.201907942.

[ref28] Wei B.; Furukawa K.; Ichikawa M.; Koyama T.; Taniguchi Y. Energy Transfer and Charge Trapping in Dye-Doped Organic Light-Emitting Diodes. Mol. Cryst. Liq. Cryst. 2005, 426, 295–302. 10.1080/15421400590891335.

[ref29] Soldano C.; Stefani A.; Turatti G.; Generali G.; Biondo V.; Basiricò L.; Ortolani L.; Morandi V.; Rizzoli R.; Veronese G. P.; Rizzoli R.; Capelli R.; Muccini M. ITO-free Organic Light Emitting Transistor Using Graphene Gate Electrode. ACS Photonics 2014, 1, 1082–1088. 10.1021/ph500289s.

[ref30] Albeltagi A.; Gallegos-Rosas K.; Soldano C. High-k Fluoropolymers Dielectrics for Low-Bias Ambipolar Organic Light Emitting Transistors (OLETs). Materials 2021, 14, 7635 10.3390/ma14247635. 34947231 PMC8704791

[ref31] Soldano C.; Generali G.; Cianci E.; Tallarida G.; Fanciulli M.; Muccini M. Engineering organic/inorganic alumina-based films as dielectrics for red organic light emitting transistors. Thin Solid Films 2016, 616, 408–414. 10.1016/j.tsf.2016.09.004.

[ref32] Moschetto S.; Benvenuti E.; Usta H.; Ozdemir R.; Facchetti A.; Muccini M.; Prosa M.; Toffanin S. Interplay between Charge Injection, Electron Transport, and Quantum Efficiency in Ambipolar Trilayer Organic Light-Emitting Transistors. Adv. Mater. Interfaces 2022, 9, 2101926 10.1002/admi.202101926.

[ref33] Liu C.; Xu Y.; Li Y.; Scheideler W.; Minari T. Critical Impact of Gate Dielectric Interfaces on the Contact Resistance of High-Performance Organic Field-Effect Transistors. J. Phys. Chem. C 2013, 117, 12337–12345. 10.1021/jp4023844.

[ref34] Kong L.; Wu J.; Li Y.; Cao F.; Wang F.; Wu Q.; Shen P.; Zhang C.; Luo Y.; Wang L.; Turyanska L.; Ding X.; Zhang J.; Zhao Y.; Yang X. Light-emitting field-effect transistors with EQE over 20% enabled by a dielectric-quantum dots-dielectric sandwich structure. Sci. Bull. 2022, 67, 529–536. 10.1016/j.scib.2021.12.013. 36546174

[ref35] Nam S.; Chaudhry M. U.; Tetzner K.; Pearson C.; Groves C.; Petty M. C.; Anthopoulos T. D.; Bradley D. D. C. Efficient and Stable Solution-Processed Organic Light-Emitting Transistors Using a High-k Dielectric. ACS Photonics 2019, 6, 3159–3165. 10.1021/acsphotonics.9b01265.

[ref36] Prosa M.; Benvenuti E.; Pasini M.; Giovanella U.; Bolognesi M.; Meazza L.; Galeotti F.; Muccini M.; Toffanin S. rganic Light-Emitting Transistors with Simultaneous Enhancement of Optical Power and External Quantum Efficiency via Conjugated Polar Polymer Interlayers. ACS Appl. Mater. Interfaces 2018, 10, 25580–25588. 10.1021/acsami.8b06466. 29984985

[ref37] McCarthy M. A.; Liu B.; Donoghue E. P.; Kravchenko I.; Kim D. Y.; So F.; Rinzler A. G. Low-Voltage, Low-Power, Organic Light-Emitting Transistors for Active Matrix Displays. Science 2011, 332, 570–573. 10.1126/science.1203052. 21527708

[ref38] Kitamura M.; Arakawa Y. Pentacene-based organic field-effect transistors. J. Phys.: Condens. Matter 2008, 20, 184011 10.1088/0953-8984/20/18/184011.

[ref39] Soldano C.; D′Alpaos R.; Generali G. Highly Efficient Red Organic Light-Emitting Transistors (OLETs) on High-k Dielectric. ACS Photonics 2017, 4, 800–805. 10.1021/acsphotonics.7b00201.

[ref40] Noh S.; Suman C. K.; Hong Y.; Lee C. Carrier conduction mechanism for phosphorescent material doped organic semiconductor. J. Appl. Phys. 2009, 105, 033709 10.1063/1.3072693.

[ref41] Sanderson S.; Philippa B.; Vamvounis G.; Burn P. L.; White R. D. Understanding charge transport in Ir(ppy)3:CBP OLED films. J. Phys. Chem. A 2019, 150, 094110 10.1063/1.5083639. 30849896

[ref42] Tonnelé C.; Stroet M.; Caron B.; Clulow A. J.; Nagiri R. C. R.; Malde A. K.; Burn P. L.; Gentle I. R.; Mark A. E.; Powell B. J. Elucidating the Spatial Arrangement of Emitter Molecules in Organic Light-Emitting Diode Films. Angew. Chem., Int. Ed. 2017, 56, 8402–8406. 10.1002/anie.201610727. 28170127

[ref43] Staroske W.; Pfeiffer M.; Leo K.; Hoffman M. Single-Step Triplet-Triplet Annihilation: An Intrinsic Limit for the High Brightness Efficiency of Phosphorescent Organic Light Emitting Diodes. Phys. Rev. Lett. 2007, 98, 197402 10.1103/PhysRevLett.98.197402. 17677659

[ref44] Reineke S.; Schwartz G.; Walzer K.; Leo K. Direct observation of host–guest triplet–triplet annihilation in phosphorescent solid mixed films. Phys. Status Solidi RRL 2009, 3, 67–69. 10.1002/pssr.200802266.

[ref45] Kalinowski J.; Stampor W.; Mężyk J.; Cocchi M.; Virgili D.; Fattori V.; Di Marco P. Quenching effects in organic electrophosphorescence. Phys. Rev. B 2002, 66, 235321 10.1103/PhysRevB.66.235321.

[ref46] Mezyk J.; Meinardi F.; Tubino R.; Cocchi M. Exciton dissociation in tris(2-phenylpyridine) iridium (III) probed by electric field-assisted time-resolved photoluminescence. Appl. Phys. Lett. 2008, 93, 093301 10.1063/1.2976782.

[ref47] Koopman W. W. A.; Toffanin S.; Natali M.; Troisi S.; Capelli R.; Biondo V.; Stefani A.; Muccini M. Mapping of Charge Distribution in Organic Field-Effect Transistors by Confocal Photoluminescence Electromodulation Microscopy. Nano Lett. 2014, 14, 1695–1700. 10.1021/nl402603c. 24611682

[ref48] Muccini M. A bright future for organic field-effect transistors. Nat. Mat. 2006, 5, 605–613. 10.1038/nmat1699. 16880804

[ref49] Melzer C.; Seggern H. V. Enlightened organic transistors. Nat. Mat. 2010, 9, 470–472. 10.1038/nmat2775. 20489698

[ref50] van Eersel H.; Bobbert P. A.; Janssen R. A. J.; Coehoorn R. Monte carlo study of efficiency roll-off of phosphorescent organic light-emitting diodes: Evidence for dominant role of triplet-polaron quenching. Appl. Phys. Lett. 2014, 105, 143303 10.1063/1.4897534.

[ref51] Giebink N. C.; Forrest S. R. Quantum efficiency roll-off at high brightness in fluorescent and phosphorescent organic light emitting diodes. Phys. Rev. B 2008, 77, 235215 10.1103/PhysRevB.77.235215.

