# Supplementary material for: TCTA:Ir(ppy)3 Green Emissive Blends in Organic Light-Emitting Transistors (OLETs)
Source: ACS Omega. 2022 Nov 18;7(48):43719–28. doi: 10.1021/acsomega.2c04718 (PMC9730476; doi:10.1021/acsomega.2c04718)
Supplement: Supplementary file 1 — ao2c04718_si_001.pdf [file ao2c04718_si_001.pdf]

Supporting Information

TCTA:Ir(ppy)$_3$ Green Emissive Blends in Organic Light-Emitting Transistors (OLETs)

Caterina Soldano*,¥, Ornella Laouadi and Katherine Gallegos-Rosas*,¥

Department of Electronics and Nanoengineering, School of Electrical Engineering, Aalto University, 02150 Espoo, Finland.

* Corresponding author(s):
katherine.gallegosrosas@aalto.fi (KGR), caterina.soldano@aalto.fi (CS)

¥ These authors contributed equally

1. Photoluminescence studies of the TCTA:Ir(ppy)$_3$ blends

Figure S1 shows the absolute PL spectra, with excitation at 403 nm at room temperature, for the different TCTA:Ir(ppy)$_3$ blends measured in the limit of same incident power and integration times (same set of data presented in Figure 1, main manuscript). PL spectra shows a quenching of the PL signal for increasing guest concentration, likely due to exciton-exciton quenching upon Ir(ppy)$_3$ clustering and formation of percolation paths. Figure S1 also shows the broadening of the spectra for increasing Ir content within the blends.

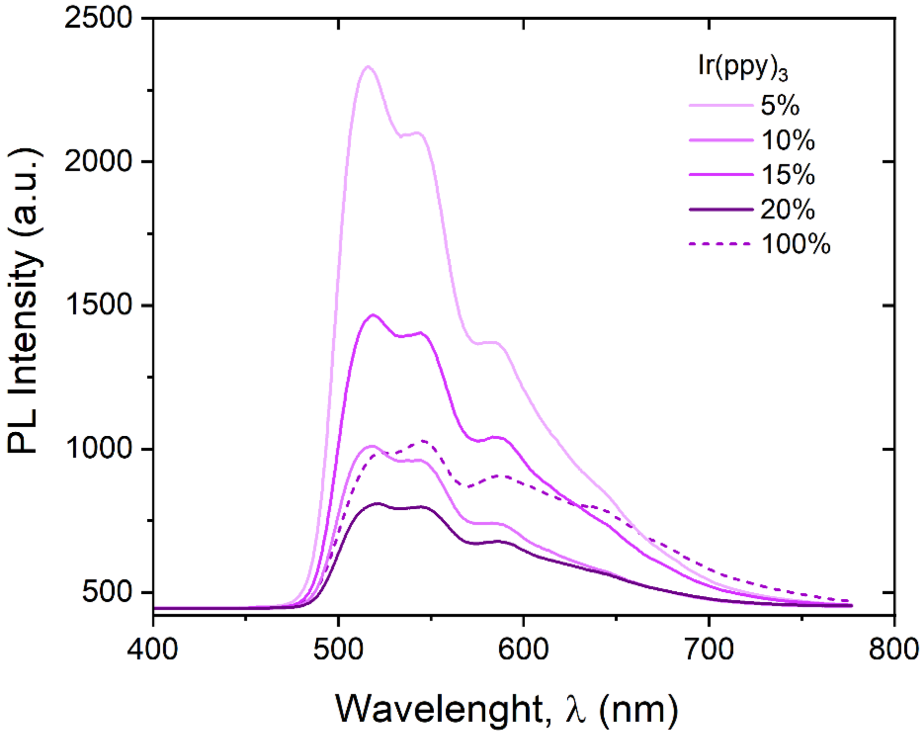

Figure S1. Absolute PL signal shows an increasing quenching of the signal for increasing guest concentration. Spectra are acquired in the limit of same incident laser wavelength, power and integration time.

Figure S2 shows a zoom of the normalized PL spectra presented in Figure 1 (main manuscript), to highlight the observed PL variation and red-shift of the main contributing peaks in the emission of the different blends (see main text for more details).

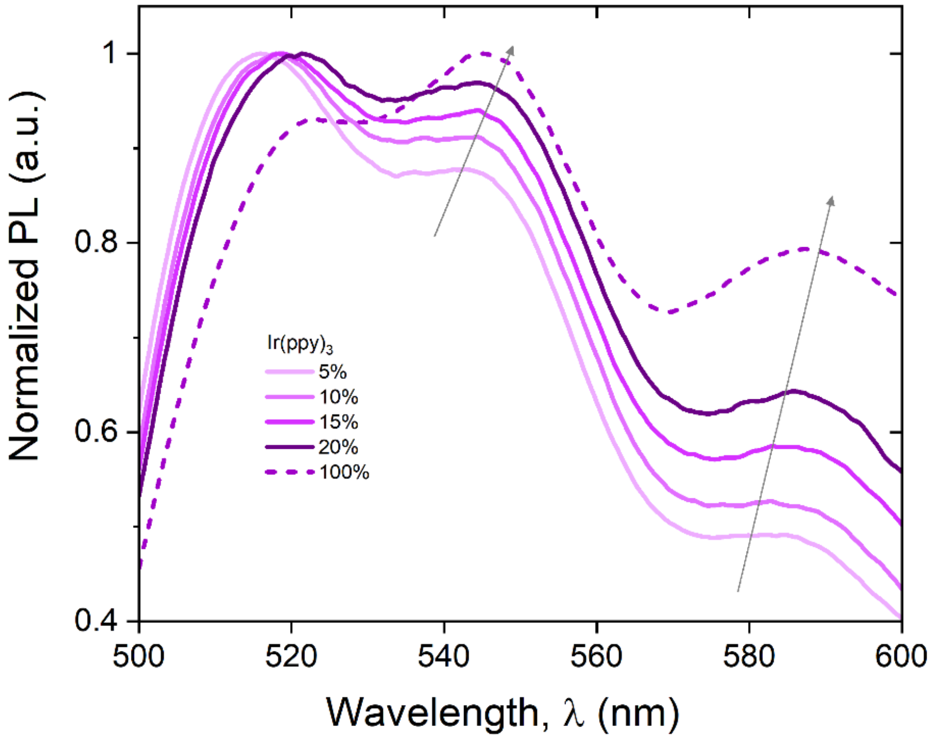

Figure S2. Zoom of the normalized PL of TCTA:Ir(ppy)$_3$ blends as function of the Ir content, showing PL variation and red-shift.

2. Hole- and electron currents in OLET devices with different TCTA:Ir(ppy)$_3$ blends

Figure S3 shows the values of *p*-type and *n*-type drain-source current as measured for OLET with different guest concentration as measured in the saturation regime (@V_{DS} = V_G= |100|V) for all devices. The current measured at the largest applied bias (|100|V) is also the maximum current recorded. A large variability is observed for the hole conduction (compared to electrons), however we do not observe any significant correlation with the guest doping concentration. This is expected being the device transport being mainly dominated by the two organic semiconductors (C8BTBT and DFH-4T). A balanced transport within the emissive blends (mobility of approximately 10^{-6} cm^2/Vs for both holes and electrons) does not contribute to the overall device currents level; however, we cannot exclude that such condition might affect the transport characteristics at the interface among the different layers. Dashed lines are guide-to-the-eye only.

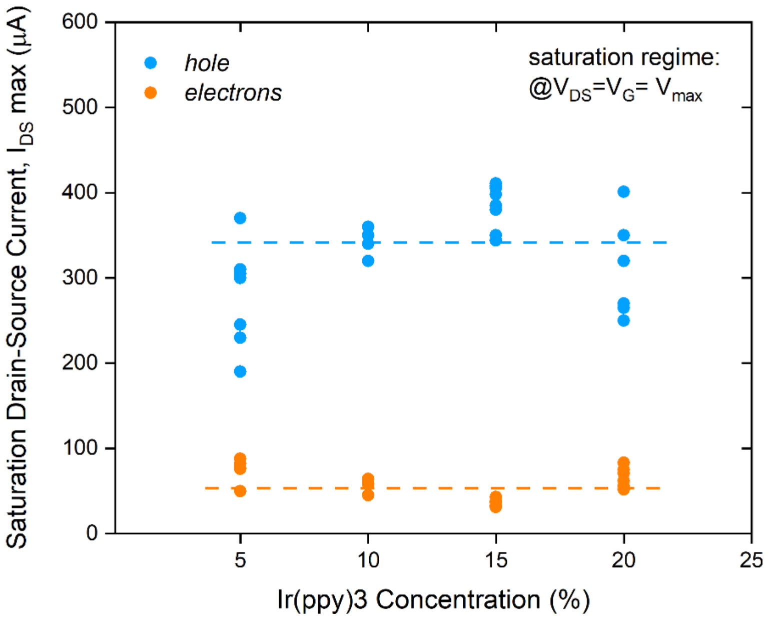

Figure S3. *p*-type and *n*-type maximum drain source current for all fabricated and studied organic light-emitting transistors with different TCTA:Ir(ppy)$_3$ blends. All values are measured in saturation regime at maximum applied bias ($V_{DS} = V_G = V_{max} = |100|$ V). Dashed lines are *guide-to-the-eye* only.

3. Optoelectronic characterization of OLET with different TCTA:Ir(ppy)$_3$ blends

Figure S4 and S5 shows the locus curves (I_{DS} *vs.* V_{DS} (=V_{GS})) for both holes and electrons for OLET devices using different Ir(ppy)$_3$ concentration (labelled accordingly in each panel). Scales for drain-source currents and light output are the same in all panels to favor an easier comparison among devices. We here note that this the light directly measured by the photodiode in direct contact with the bottom of the substrate. While a large increase is observed for TCTA:Ir(ppy)$_3$ 10% in terms of light output, however we do not observe any correlation with the guest doping concentration. We also note that our organic light emitting devices are being dominated by hole transport (*i.e.* C8BTBT). We observe no light for the case of electron transport.

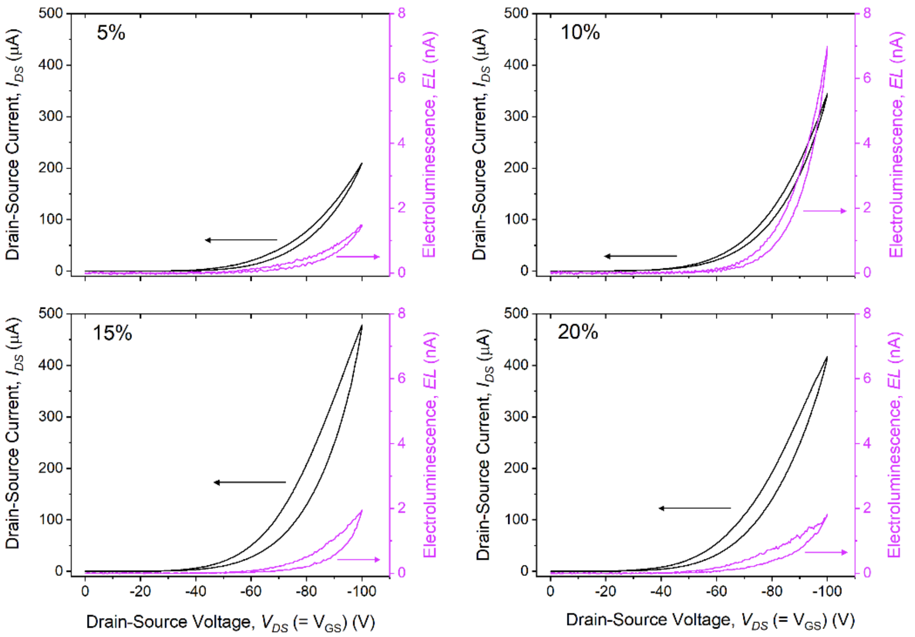

Figure S4. *p*-type locus curves (I_{DS} *vs.* V_{DS}=V_{GS}) for organic light-emitting transistors using different TCTA:Ir(ppy)$_3$ blends.

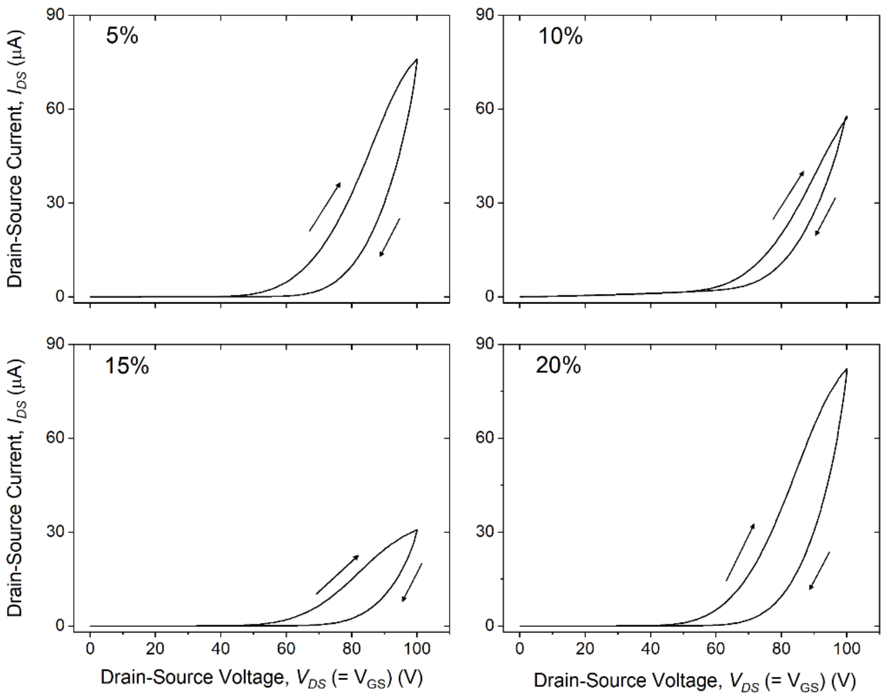

Figure S5. *n*-type locus curves (I_{DS} *vs.* V_{DS}=V_{GS}) for organic light-emitting transistors using different TCTA:Ir(ppy)$_3$ blends. No light is observed.
